# Nonischemic and normothermic beating cardiac xenotransplantation

**DOI:** 10.3389/frtra.2026.1984456

**Published:** 2026-10-30

**Authors:** Daniel H. Wolbrom, Nobutoshi Matsumura, Morgan K. Moroi, Ishit Chauhan, Sho Fujiwara, Susan Qudus, Michael Brewer, Dilrukshi Ekanayake-Alper, Dominik Hajosi, Lorne Schweitzer, Koji Takeda, Richard J. Levy, Stephan M. Juergensen, Oliver Barry, Miroslav Sekulic, David H. Sachs, Megan Sykes, Greg Nowak, Andrew B. Goldstone

**Affiliations:** 1 Columbia Center for Translational Immunology, Columbia University, New York, NY, United States; 2 Department of Surgery, Columbia University, New York, NY, United States; 3 Department of Anesthesiology, Columbia University, New York, NY, United States; 4 Division of Pediatric Cardiology, Columbia University, New York, NY, United States; 5 Department of Pathology and Cell Biology, Columbia University, New York, NY, United States; 6 Department of Medicine, Columbia University, New York, NY, United States; 7 Department of Microbiology and Immunology, Columbia University, New York, NY, United States

**Keywords:** beating heart transplant, cardiac xenotransplantation, *ex vivo* perfusion, genetically modified pigs, heart transplant, pig-to-nonhuman primate transplant

## Abstract

Cardiac xenotransplantation—the transplantation of a heart from one species to another- offers a clinically viable solution to the organ shortage problem. Despite significant advances in genetic engineering technologies and new immunosuppressive medications such as co-stimulatory blockade, bringing cardiac xenotransplantation to the clinic still has several challenges. Perioperative cardiac xenograft dysfunction is a major limitation to long-term xenograft survival and is attributed to the ischemia-reperfusion damage associated with the procurement and transplant of the porcine heart. This dysfunction can be reduced by utilizing commercial *ex vivo* perfusion devices which limit the cold ischemia time. Unfortunately, these devices still require an obligatory ischemic period during the initial arrest with cardioplegia and subsequent explanting of the organ, and then again during the implantation of the heart. These commercial devices also do not support smaller pediatric sized hearts, reducing the pool of recipients eligible for xenografts. We sought to establish a novel method of explanting and orthotopically implanting beating donor hearts from genetically modified pigs without any ischemia, while maintaining normothermic perfusion in a pig-to-nonhuman primate model. Eliminating cold and warm ischemia has never previously been achieved in cardiac xenotransplantation and has important implications for bringing it to the clinic, as well as broader translatability in allotransplantation. We performed a proof-of-concept experiment utilizing our novel technique in a non-survival model and then performed a successful experiment with nearly 8 weeks of survival of a single *GGTA1* knockout pig heart. Our initial data suggest that utilization of a normothermic coronary perfusion circuit in cardiac xenotransplantation is feasible and safe and may be a plausible method towards achieving survival.

## Introduction

While the incidence of heart failure is expected to continue to increase by as much as 40% by 2050 (1), the supply of allogeneic hearts has not kept pace with the rising demand (2). In the pediatric population suffering from congenital heart disease, this burden is even greater, as the options for this population are limited (3). In fact, the heart transplant waitlist mortality for children is ∼20% for those not transplanted by one year (4). Despite efforts to increase the supply of allogeneic hearts by increasing the utilization of high-risk donors (5) and donation after circulatory death (6), the supply of suitable organs still does not meet the demand, and an alternative source of donor hearts is urgently needed. The ability to transplant porcine hearts into humans, termed cardiac xenotransplantation, is a potential solution to the organ shortage crisis (7, 8). The technology to produce genetically engineered pigs, which can be bred to near limitless numbers in biologically secure facilities, offers a potential paradigm shift in the field of heart transplantation (9). Several advancements over the last two decades, such as technologies to genetically engineer pigs using Clustered Regularly Interspaced Short Palindromic Repeats (CRISPR)-Cas9 (10), coupled with novel immunosuppressive therapies such as co-stimulatory blockade (11) and complement inhibitors (12, 13) have brought cardiac xenotransplantation to the brink of clinical application (United Therapeutics has received FDA approval to begin enrolling two patients to a clinical trial utilizing their 10-gene edited porcine heart) (14). However, additional research is needed before it can be safely added to the armamentarium of treatment options for those with heart failure.

One of the biggest hurdles in the clinical application of cardiac xenotransplantation is the ischemia-reperfusion (IR) injury associated with these xenografts (15). In allotransplantation, grafts are subject to the negative sequelae of IR injury, mediated by complement activation, sterile inflammation, and endothelial activation (16, 17). These pathways are more pronounced in cardiac xenotransplantation because species-dependent molecular incompatibilities provoke a profound immunologic response, resulting in more severe clinical manifestations of IR injury (18). In cardiac xenotransplantation, this phenomenon is termed perioperative xenograft dysfunction (PCXD) and is a significant source of morbidity and mortality in animal experiments (19). PCXD has been observed to occur in 40%–60% of orthotopic cardiac xenotransplantation experiments in nonhuman primates (NHP) (15). While the precise mechanism of this phenomenon is still unclear, it is generally considered to be defined as the rapidly developing loss of cardiac function within the first 48 h of implantation and occurs independently of hyperacute rejection (20). In a series of NHP experiments by Langin et al., it was demonstrated that in the setting of a relatively short ischemic time (mean of 123 min), survival in 3 animals was <48 h and <30 days in the other 2 animals, with all 5 animals lost due to severe systolic left heart failure. To eliminate PCXD and extend heart xenograft survival, their group devised methods to reduce cold ischemia time by perfusing the xenograft at 8 degrees Celsius after explanting the heart from the pig donor (21). Despite the utilization of *ex vivo* perfusion devices, the longest survival of an orthotopically transplanted pig heart into a NHP after *ex vivo* perfusion is 9 months, far shorter than what typical allogeneic heart transplants can survive (22).

More recently, Cleveland et al. demonstrated prolonged survival in a series of fifteen orthotopic cardiac xenotransplantation in NHPs without *ex vivo* perfusion, with the longest survival of two years (734 days) and a mean of 41 days. While these experiments resulted in the longest surviving NHP after cardiac xenotransplantation, 40% (6 out of 15) of the animals in this study expired in <7 days, often from primary graft dysfunction and systemic inflammatory responses (23). Cleveland et al. do not describe this shortened survival in 40% of the subjects as PCXD, although these results are not dissimilar to what McGregor et al. described as PCXD in their experience (19, 20). The results presented by Cleveland's group are a significant step forward in demonstrating long term survival of cardiac xenografts in the orthotopic position, but it is unclear if utilizing a shortened cold ischemic preservation period without *ex vivo* perfusion result in a phenomenon similar to the PCXD described in other studies (15, 19, 20, 24).

To improve upon the duration of survival in cardiac xenotransplantation and reduce the rates of PCXD, we sought to develop and study a novel technique of heart transplantation utilizing genetically modified pig hearts in a NHP experimental model. Our hypothesis is that by eliminating ischemia-reperfusion and keeping the heart perfused at normothermia and invariably beating, instead of just reducing the duration of ischemia, we can achieve decreased postoperative graft dysfunction and prolong length of survival. The purpose of this manuscript is to describe our novel technique and demonstrate the feasibility of performing such a procedure. Eventually, we believe this technique may accelerate further progress in cardiac xenotransplantation.

## Methods and materials

### Non-survival proof-of-concept experiment

To demonstrate the feasibility of performing a beating heart xenotransplantation in a nonhuman primate model, we first sought to conduct our experiment in a non-survival model, with a plan to euthanize the recipient one hour after separating from cardiopulmonary bypass. As such, we utilized a 16 kg alpha-1,3 galactosyltransferase knockout (*GGTA1* KO) pig (*Chazy, NY, USA*) from the Sachs herd of miniature swine, and a 24 kg male baboon (*Papio anubis*) purchased from The University of Texas MD Anderson Cancer Center (*Houston, TX, USA*). Since this was intended as a non-survival experiment, the baboon recipient had high pre-transplant non-Gal anti-pig antibody and cytotoxicity levels (25). Additionally, no induction immunosuppression protocol was used for the same reason.

### Selection of donor and recipient for survival transplant

A juvenile *GGTA1* KO pig (*Chazy, NY, USA*) from the Sachs herd of miniature swine, weighing 5.0 kg and negative for porcine cytomegalovirus (pCMV) was selected as the donor for a 5.3 kg male baboon (*Papio anubis*) purchased from The University of Texas MD Anderson Cancer Center (*Houston, TX, USA*). The recipient baboon was screened prior to transplant for non-Gal anti-pig antibodies and cytotoxicity, the detailed methods of which are described elsewhere (26). Briefly, we selected the recipient with low cytotoxicity of preformed natural antibody against *GGTA1* null pig peripheral blood mononuclear cells (PBMCs) at 1:4 ratio of effector to targets (46%). Anti-non-Gal IgM and IgG serum antibodies were evaluated using *GGTA1* null PBMCs and detected by indirect FACS using PE-conjugated goat anti-human-IgM antibody (*clone MHM-88*) or AF647-conjugated goat anti-human-IgG antibody (*clone MHL-38*). Data were expressed as median fluorescence intensity (MFI), and then an index was calculated by (Experimental median fluorescence intensity—negative control MFI)/(positive control MFI—negative Control MFI) × 100). The recipient was selected to have an IgG index <10 and IgM index <20.

All animal care, procedures, and surgeries were performed in accordance with the United States Department of Agriculture (USDA) and National Institutes of Health (NIH) guidelines, with approval from Columbia University's Institutional Animal Care and Use Committee (IACUC protocol number AC-AABY7650 and AC-AABN1550). All animals were housed at the Institute of Comparative Medicine (ICM) at Columbia University, which provided veterinarian and husbandry care. Columbia University is an Association for Assessment and Accreditation of Laboratory Animal Care accredited institution.

### Preoperative central venous catheter and arterial line placement

Placing indwelling central venous lines 7–14 days preoperatively is essential to provide intravenous medications, including the immunosuppression and medication needed to support hemodynamics in the immediate postoperative state. Two central venous catheters are placed, one in the left internal jugular vein and one in the left femoral vein. The left jugular vein is used to preserve the right jugular vein which will be used for access to perform right heart catheterizations and biopsies. To ensure the animal cannot reach and damage the lines, the lines are tunneled subcutaneously to the back of the animal to exit the skin between the scapulas. The lines are then connected via a system of clear Tygon tubing (*Norton Performance Plastics, Akron, OH, USA*) that is protected with 1.0-to-1.5-meter aluminum tether that is attached to a rigid swivel at the top of the cage. This facilitates access to the lines from outside the cage. The animal is then placed in a mesh jacket (*Lomir Biomedical Inc., Malone, NY, USA)* to ensure the lines and connection point to the tether are protected from damage.

On the day of the transplant, the arterial line is placed in the left carotid artery to facilitate close monitoring of the animal via blood draws, blood gases, and continuous intra-arterial pressure monitoring. We prefer to leave the arterial line placement until the day of transplant to reduce the likelihood of complications related to inadvertent dislodgement of the arterial line, which could be lethal. Briefly, the animal is positioned supine to access the left neck. The prior incision is re-opened, and the carotid artery is identified via blunt dissection. The artery is encircled to give distal and proximal control, and then the distal end if ligated with a 0–0 silk tie to prevent back bleeding. Next, the arteriotomy is created, and a 6.5 Fr single lumen Hickman central line (*Bard Access Systems, Inc., Salt Lake City, UT, USA*) is advanced 3–4 cm. The size of the arterial catheter can be adjusted based on the size of the animal, but typically a 6.5 Fr is the largest catheter that can be used in a 5 kg NHP. A silk tie is used over the proximal end of the carotid artery to secure the catheter, and then the distal portion is secured with another silk tie to provide two points of fixation. The catheter is then tunneled to the back in the similar fashion as the central venous catheters, and the neck incision closed in layers.

### Donor heart procurement and *ex vivo* perfusion

The donor pig was prepped and draped in standard surgical fashion in the supine position. The anatomy of the pig heart is similar to primates, with several subtle differences outlined previously (27). The sternum was opened with a pair of heavy sheers, and a retractor placed to visualize the pericardial sac. Next, the pericardium was opened, and the pericardial cradle created. The donor heart was dissected in a similar manner to standard heart procurement methods. In order to remove the donor heart while beating, the pig heart was placed on a normothermic coronary perfusion pump primed with the donor pig's heparinized blood, on a Spectrum Quantum Charlotte frame (*Spectrum Medical, Fort Mill, SC, US*) utilizing a D100 Oxygenator (*LivaNova, Houston, TX, USA*) and tubing pack (1/8” × 3/16” loop) with 12 Fr malleable drainage/inflow cannulas in the right atrium (RA) and a 12 Fr vent in left ventricle (LV) apex (*Medtronic, Minneapolis, MN, USA*), and 6 Fr DLP (*Medtronic, Minneapolis, MN, USA*) outflow cannula in the proximal ascending aorta. The RA and LV cannulas were connected to a common line via a Y-connector. The coronary perfusion pump utilized a reservoir to allow a pump sucker to collect potential blood spilled from the donor heart and recirculate back into the circuit. Total priming of circuit was 135 mL, which included 50 mL 25% albumin, 5 mEq sodium bicarbonate, 500 units of heparin, and 80 mL of Plasmalyte A. Flow rates were kept in a range of 60–100 mL/min. The superior vena cava (SVC) and inferior vena cava (IVC) were ligated, the aorta was clamped, and then the heart was explanted while maintaining antegrade flow into the proximal aorta to provide coronary blood flow (Figure 1). Following successful explant of the donor heart, it was placed in a sterile surgical bucket with a pump sucker to recirculate blood into the circuit. The donor heart was then transported carefully to an adjoining operating room with the perfusion circuit and adjoining cannulas, taking care not to dislodge or kink the catheters. The temperature was carefully maintained on the coronary perfusion pump to maintain normothermia. The pig heart was then brought onto the surgical field of the recipient and prepared for implantation.

**Figure 1:**
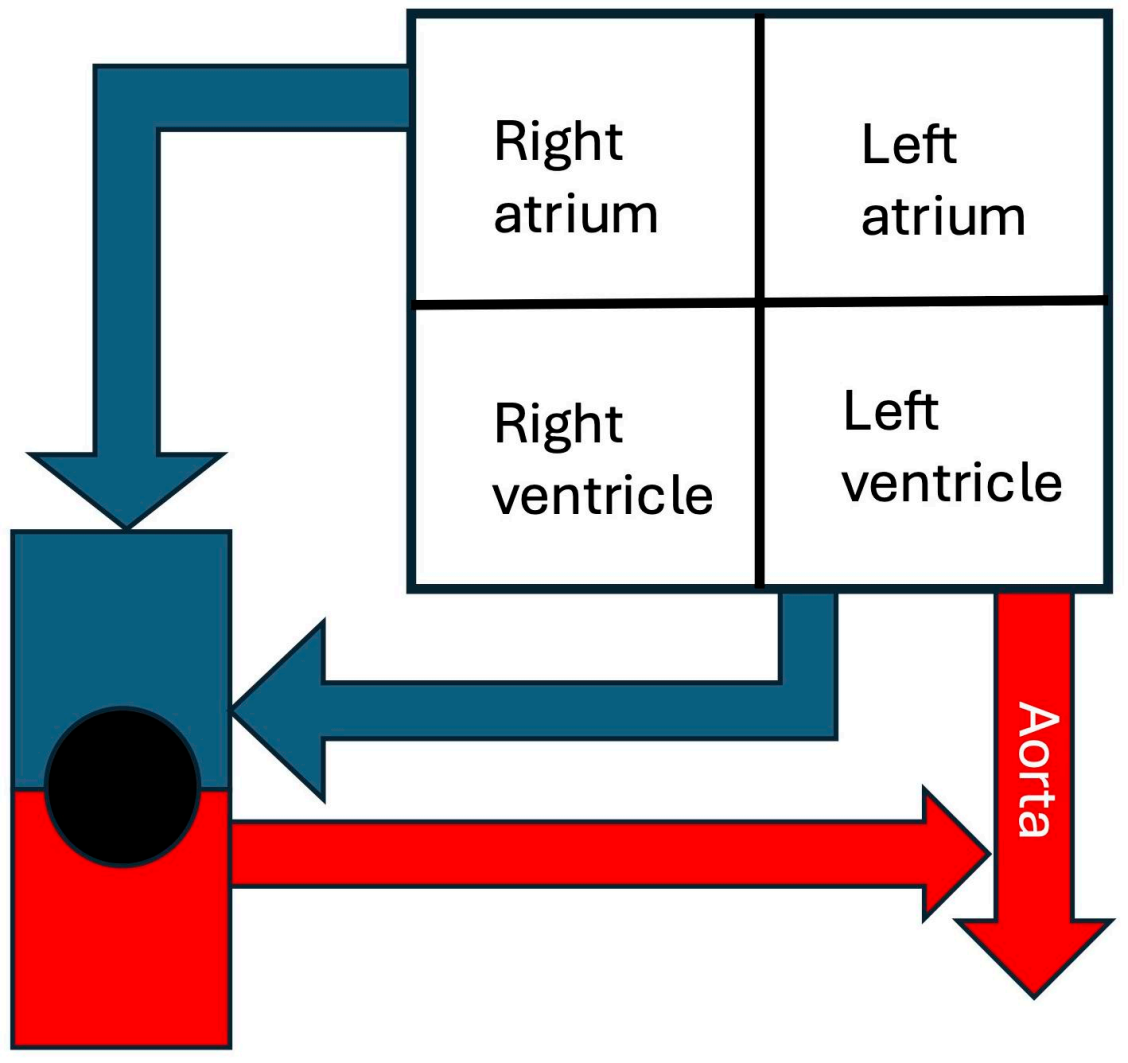
Schematic of *ex-vivo* perfusion.

### Cardiopulmonary bypass circuit and donor heart implantation

Standard midline sternotomy was performed. After administration of 3,000 units of heparin, the bypass was configured for our 5.3 kg baboon recipient on a Jostra HL 20 Heart Lung machine (*Soma Technology, Bloomfield, CT, USA*) with a Terumo FX05 Oxygenator (*Terumo Cardiovascular, Ann Arbor, MI, USA*) with custom Liva Nova tubing pack (3/16” × ¼” loop) (*LivaNova PLC, London, UK*). The aortic cannula selected was a 10 Fr Biomedicus and a 12 Fr and 16 Fr venous cannula with a metal right angle tip (*Medtronic, Minneapolis, MN, USA*). A 10 Fr vent was used as well (*Medtronic, Minneapolis, MN, USA*). The total priming solution was 220 mL and consisted of 50 mL 25% albumin, 8 mEq sodium bicarbonate, 900 units of heparin, and 65 mL of Plasmalyte A and 100 mL of irradiated (30 Gy) and washed baboon whole blood from a 3rd party blood donor with known low levels of non-Gal anti-pig antibody levels. Full cardiopulmonary bypass (CPB) was initiated. The heart was then explanted by transecting the aorta, pulmonary artery, left atrium (including the appendage), the SVC, and the right atrium at the IVC junction. When the beating donor heart was brought into the field, we first performed the left atrial anastomosis with 4–0 polypropylene suture (*Prolene®, Ethicon, Somerville, NJ, USA*). A DLP vent was placed in the LV via the left atrial suture line. The aortic anastomosis was performed with a single layer of 5–0 polypropylene suture (*Prolene®, Ethicon, Somerville, NJ, USA*). After completing the aortic anastomosis and deairing the left side of the heart, the coronary perfusion circuit was turned off and the cross-clamp removed to begin recipient reperfusion of the xenograft. The pulmonary artery and then right atrial anastomosis were then performed with a 5–0 polypropylene suture (*Prolene®, Ethicon, Somerville, NJ, USA*). Following successful separation from cardiopulmonary bypass, heparin was reversed with protamine, hemostasis achieved, and a soft Blake drain chest tube placed. The sternum was closed in standard fashion with 0–0 polydioxanone suture (*PDS™ II, Ethicon, Somerville, NJ, USA*), and the muscle and skin closed in layers.

### Immunosuppression

Our induction immunosuppression regimen for the survival model consisted of the following: anti-CD20 mAb (*Rituxan®, Genentech, Inc., South San Francisco, CA, USA*) at 10 mg/kg on day −6 and day 0, rabbit anti-thymocyte globulin (rATG*; Thymoglobulin®, Sanofi, France*) at 10 mg/kg on day −3 and −2, and methylprednisone (*Solu-Medrol; Pfizer Inc., New York, NY, USA*) at 20 mg/kg on day 0 and then tapered over the first week. Additionally, anti-C5 mAb was administered weekly for 7 doses, starting at 30 mg/kg on day 0, and then 10 mg/kg thereafter. Mycophenolate mofetil *(*MMF*; CellCept®, Genentech, South San Francisco, CA, USA*) was administered daily via IV infusion starting on day −6 at 110 mg/kg. Anti-CD40 mAb (Clone 2C10, NIH nonhuman primate resource), 50 mg/kg was given days 2, 6, 8, 10, 12, tapered to 20 mg/kg days 14, 16, 18, 21, 24, 27, 30, then twice per week from day 30 to the endpoint. Recipient was scheduled to receive rapamycin (0.1 mg/kg daily) starting day 30, or sooner if left ventricular hypertrophy was observed to prevent graft growth but was delayed until day 43 due to infectious concerns (Figure 2*)*.

**Figure 2:**
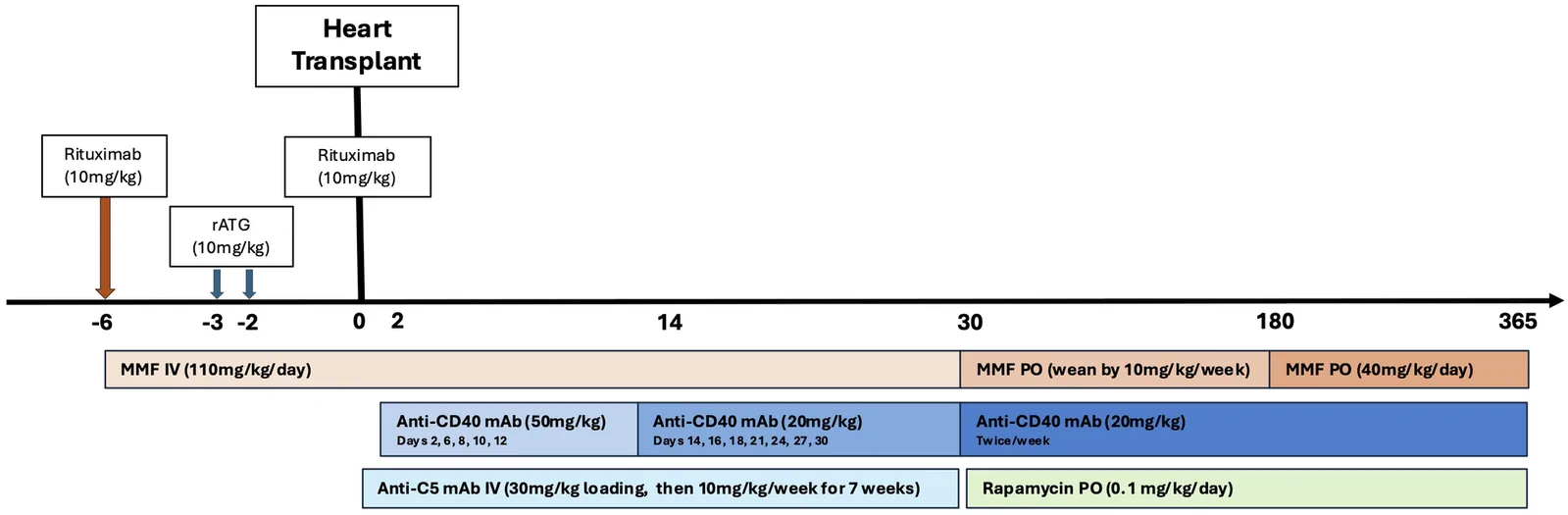
Immunosuppression regimen.

### Transthoracic echocardiography

Echocardiography was performed utilizing standard pediatric echocardiographic imaging technique and measurement methods (28). Given cardiac position differences in baboons relative to humans (29) imaging planes were modified as necessary to obtain structural and functional metrics as described. Left ventricular function and volumetrics were obtained using M-mode and 5/6 area-length methods. Cardiac index was estimated by calculation of stroke volume using left ventricular outflow tract volume-time integral, aortic valve diameter, multiplied by coinciding heart rate, indexed to BSA on the date of the exam. Intraoperative imaging was performed by epicardial echocardiography, and all subsequent transthoracic studies were performed sedated. The frequency of the transthoracic echocardiograms was at least bi-weekly, or more frequently if the clinical situation dictated.

### Right heart catheterization and biopsies

A right heart catheterization was performed after the subject was safely under anesthesia and intubated. The subject's right neck was shaved and cleaned, then prepped under sterile conditions. Using ultrasound guidance, venous access was obtained in the right internal jugular vein (RIJV) using a 21 g needle and standard 0.018” guidewire. Wire position in the RIJV was confirmed by ultrasound and fluoroscopy. The wire was exchanged for a 0.025” wire using the micropuncture dilator and a 5 Fr sheath was then advanced to the right atrium (RA). A 5 Fr balloon wedge catheter was then placed into the RA. Pressures were then measured in the RA, right ventricle (RV), pulmonary artery (PA) and a pulmonary capillary wedge pressure (PCWP) was also obtained. A blood sample was obtained in the PA for calculations. The 5 Fr sheath was then advanced over the balloon wedge catheter and positioned into the RV and the wedge catheter was then removed. Biopsies were obtained in the RV using a 5 Fr bioptome under fluoroscopic guidance. After biopsies were obtained, the sheath was safely removed and manual hemostasis achieved without issue. Calculations for flows were made using the Fick method. The subject's oxygen consumption (VO2) was assumed at 170 mL/kg/min and systemic PaO2 was assumed to be 500 mmHg with the subject intubated and breathing 100% FiO2 during the cath. The subject's hemoglobin and PA saturation were directly measured. The frequency of the right heart catheterization and biopsy was at least bi-weekly, or more frequently if the clinical situation dictated.

### Postoperative management

After closure of the sternum and skin, the baboon was kept in the operating room and intubated to maintain intensive care unit conditions. Specifically, chest tube output, blood pressure and heart rate, and arterial blood gases were monitored continuously over the next 24 h. Continuous epinephrine infusion was the pressor of choice to maintain a mean arterial blood pressure (MAP) of >55 mmHg. After 24 h and with acceptable hemodynamic data and minimal chest tube drainage, the recipient was weaned from anesthesia and extubated to room air. Following extubation and recovery, the animal was returned to the monitored housing unit, which consists of USDA certified cages. These cages are designed to have a tether system in place to ensure central venous and arterial line access and monitoring. This facilitates the close monitoring of the baboon visually as well as with hemodynamic monitoring of blood pressures and heart rates. Additionally, morning and evening labs were collected, and arterial blood gases every 8–12 h via the arterial line.

In the immediate postoperative period, dobutamine was the inotrope of choice to maintain a MAP of >55 mmHg. Additionally, aspirin PO dosed at 4 mg/kg/day was administered once all concerns for bleeding subsided, in addition to a continuous heparin infusion of 240–300 units/kg/day and epoprostenol 30 ng/kg/min continuously to prevent thrombosis for 14 days. Due to the blood pressure incompatibility between swine and primate, blood pressures were maintained in the recipient on the lower side via twice daily dosing of PO propranolol, which was titrated to maintain MAPs <65 mmHg (30, 31). Daily basic metabolic panels were obtained in the postoperative period, and all electrolyte abnormalities were identified and repleted as needed.

The arterial line was maintained for 7 days postoperatively and removed via a small neck incision and ligation of the carotid artery to ensure hemostasis. Hemodynamic monitoring was accomplished thereafter with noninvasive blood pressure measurements during planned cage side sedations. Additional clinical monitoring was performed via venous blood draws from the indwelling central venous lines and clinical observation, specifically assessing for edema, appetite, activity, weight loss, urine output, and body condition scoring. Biochemistry was obtained with the Heska Element DC5X (*Heska Corporation, Loveland, CO, USA*) and complete blood count with the Heska Element HT5 (*Heska Corporation, Loveland, CO, USA*).

Baboon CMV (bCMV) viremia, which is a common complication following organ transplantation in NHPs was carefully monitored with weekly qPCR to monitor for viral replication. In the case of active viremia, ganciclovir 12.5 mg/day would be administered to treat the animal until the viremia resolved, but this did not occur. Prophylactic piperacillin and tazobactam was administered intravenously to the recipient from the time of placement of the indwelling central line to prevent line or wound infections. Our group has observed liberal use of prophylactic antibiotics in NHP experiments to be essential since maintaining a clean environment is difficult. Upon clinical suspicion of an infection, either by clinical deterioration not related to cardiac dysfunction or laboratory data indicating an infection, escalation of antibiotics was performed in consultation with experts in transplant infectious disease medicine.

## Results

### Non-survival proof-of-concept experiment

The recipient baboon was placed on cardiopulmonary bypass in standard fashion, and the donor porcine heart was placed on the *ex vivo* perfusion circuit, as described above (Figure 1). The time on the perfusion circuit was 34 min. The donor heart remained in sinus rhythm while on the circuit for the duration of *ex vivo* perfusion. The donor heart was implanted into the recipient, as described above. The recipient baboon was weaned off cardiopulmonary bypass in sinus rhythm on no inotropes or vasopressors. Echocardiography was performed 60 min after separation from cardiopulmonary bypass and demonstrated normal qualitative RV systolic function, mildly decreased LV systolic function (LV shortening fraction 24%–25%), trivial MR, and mild tricuspid regurgitation (Figure 3*)*. Invasive pressure monitoring demonstrated a RA pressure pf 6 mmHg and PA pressure of 32/12 mmHg 60 min post separation from cardiopulmonary bypass. The xenograft provided life-sustaining cardiac output with an SvO2 of 77% and arterial blood gas (ABG) of pH 7.48, pCO2 22.2 mmHg, pO2 234.8 mmHg, and HCO3- 16.6 mmol/L. Histology demonstrated no morphologic evidence of hyperacute antibody-mediated rejection (AMR) or acute T cell-mediated rejection, ischemic injury, or edema (Figure 4).

**Figure 3:**
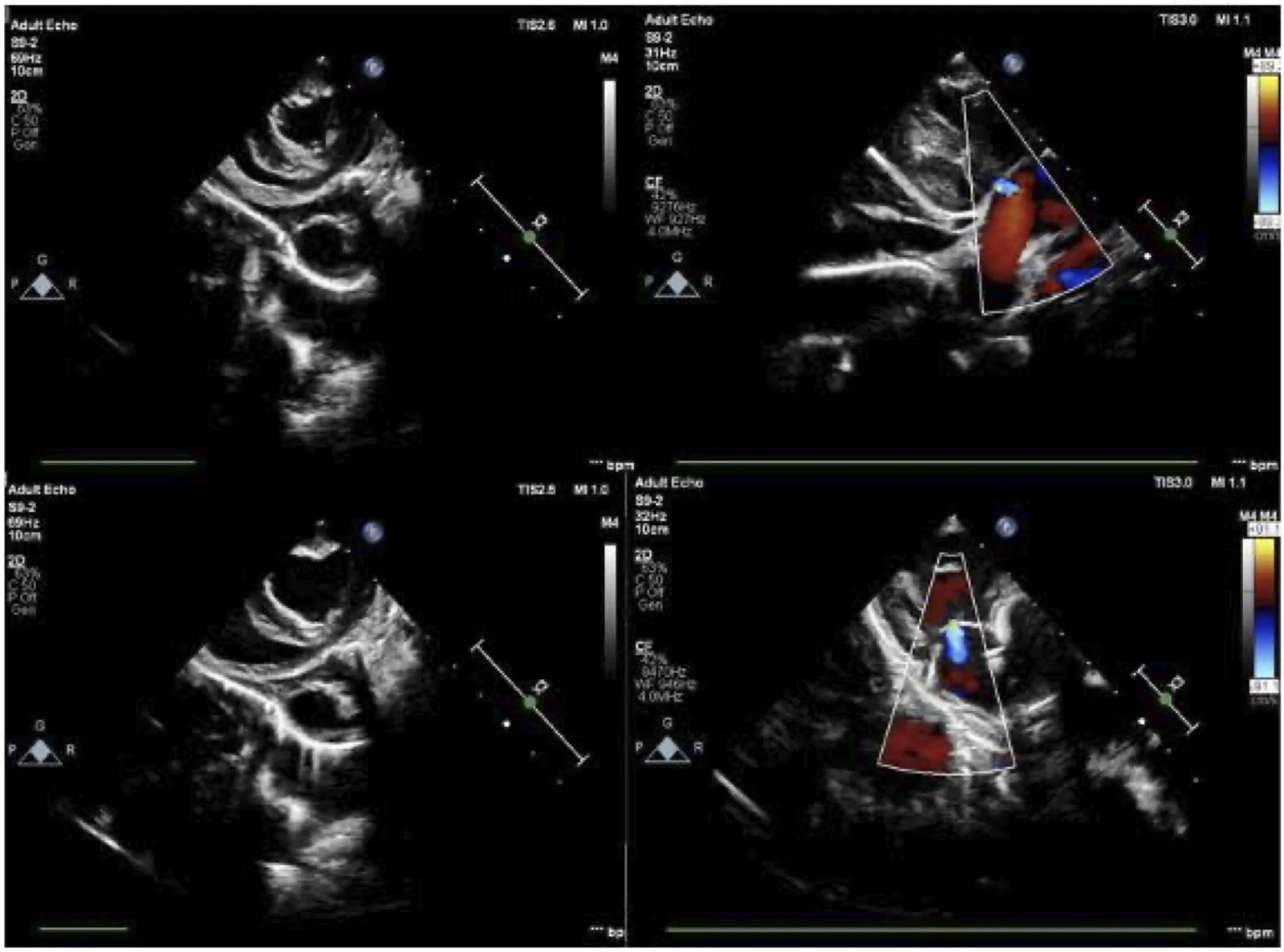
Echocardiography 60 min post separation from bypass.

**Figure 4:**
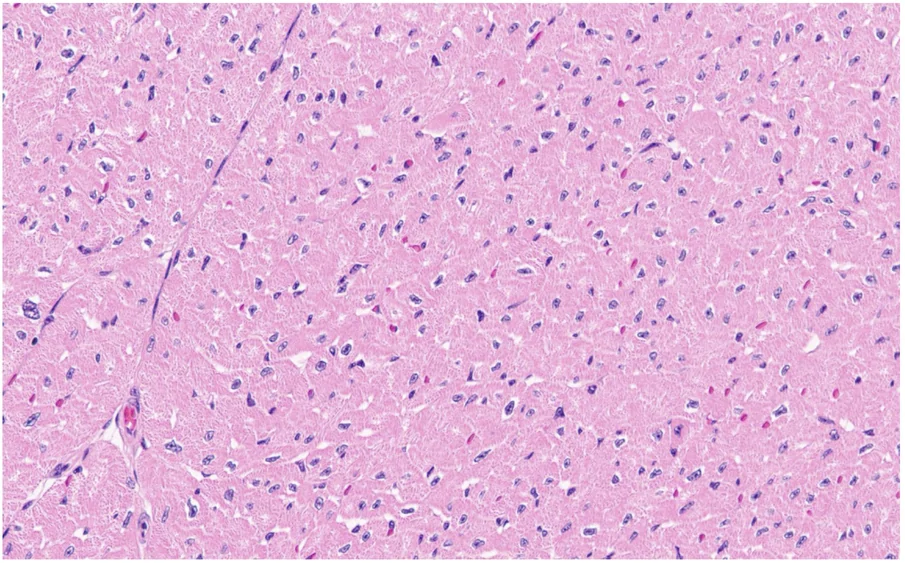
Section of myocardium stained with H&E.

### Operative survival

The 5.3 kg recipient baboon was placed on the cardiopulmonary bypass circuit successfully using the methods described above. CPB was set with a flow of 0.9–1.05 L per minute, sweep of 35–40 L per minute, and FiO2 of 75%–80%. Additionally, Sevoflurane was administered at a concentration of 2%–3% for inhalational anesthesia. After the recipient native heart was explanted in the manner described above, attention was then turned to the porcine donor heart. The porcine donor heart was explanted from the donor after securing the xenograft to the *ex vivo* perfusion device in a manner described above (Figure 1). The xenograft was transported sterilely to an adjoining operating room while beating at normothermia while connected to our *ex vivo* perfusion device (Figure 5). The beating donor heart was implanted in the manner described above, with a total time on the *ex vivo* circuit of 41 min. With the donor heart implanted successfully in the manner described above, the recipient was weaned from CPB, after a total run time of 2 h and 25 min. The hematocrit of the cardiopulmonary bypass circuit was initially 18% despite priming the circuit with 100 mL of donor pig blood. Therefore, 250 mL of circuit volume was removed via the hemoconcentrator during the procedure to increase the hematocrit. The peak lactate on CPB was 2.6 immediately after reperfusion of the xenograft but trended down afterwards. The recipient baboon was kept sedated and intubated in the operating room for 24 h to ensure close hemodynamic monitoring and support.

**Figure 5:**
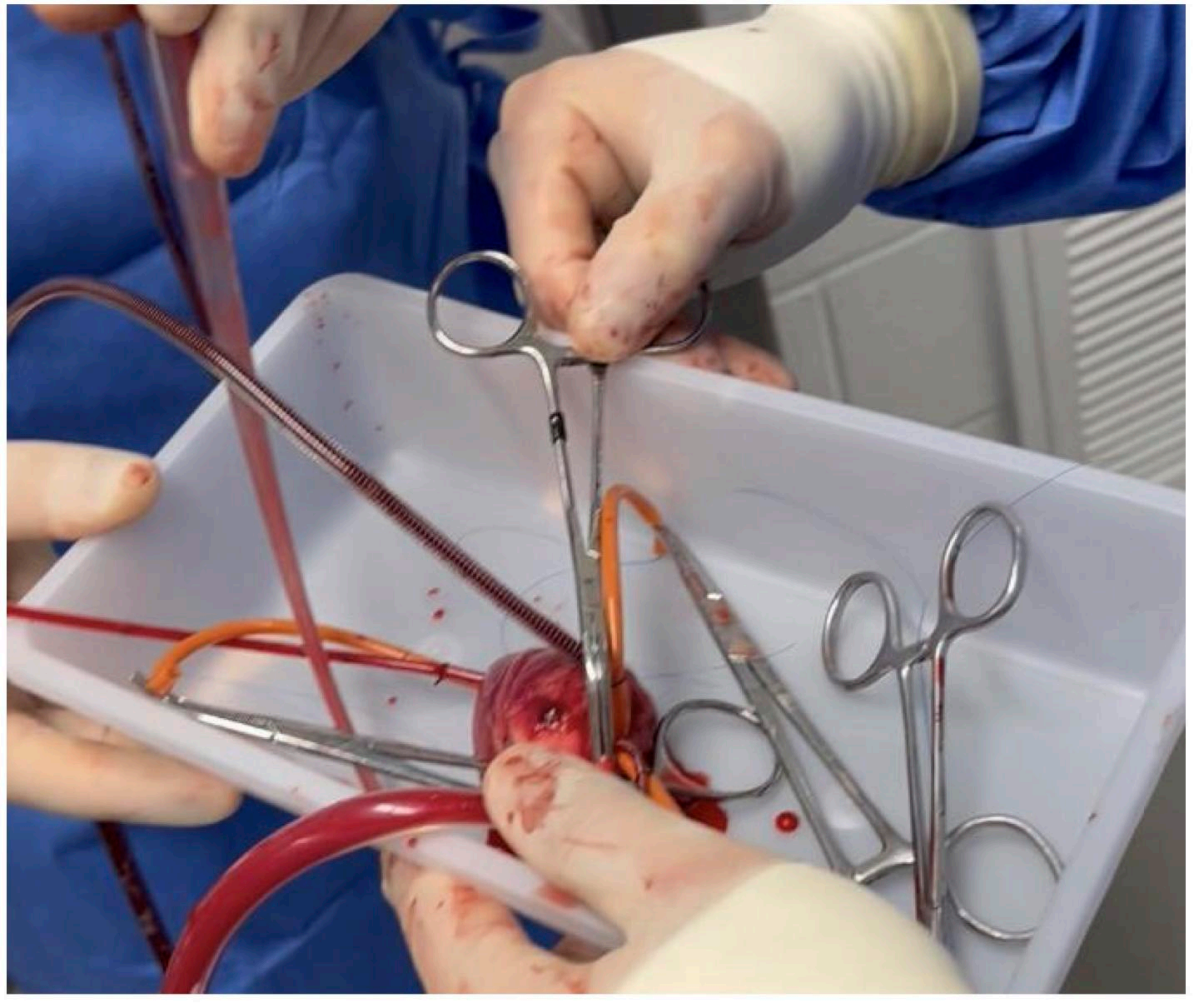
Image of *ex-vivo* beating porcine heart.

### Postoperative course

In the immediate postoperative period after closure of the sternum, the subject required inotropic support with dobutamine to maintain acceptable MAP >55 mmHg. Serum lactate was also elevated in the initial postoperative period, indicating hypoperfusion, peaking around 12 mmol/L, and trending down with adequate resuscitation and hemodynamic support. This was further supported by echocardiography in the immediate postoperative period, which demonstrated an LV ejection fraction (EF) of 15.8% and LV fractional shortening of 6.8%, moderate mitral regurgitation (MR), and moderate-to-severe tricuspid regurgitation (TR). 12–14 h postoperatively, inotropy support with dobutamine had been substantially weaned, and echocardiography improved with a LV EF of 50%, improvement in the MR and no change in TR. The recipient was started on epoprostenol and heparin drips per protocol at that point to prevent thrombotic complications, and chest tube output increased. The heparin was stopped, and an additional 60 mL of irradiated 3rd party baboon blood was transfused. Over the next several hours, the chest tube output slowed down, the hematocrit was stable, and it was decided to wean the recipient from sedation and extubate. The animal was successfully extubated and returned to the special housing unit for close monitoring and observation. For the next 7 days, the animal was closely monitored via an indwelling arterial line to monitor the blood pressure continuously and in real time, and the blood gas was measured every 6–12 h (Figure 6). On POD 1, the animal was started on a nicardipine infusion to maintain MAPs in the 55–65 mmHg range and transitioned to PO propranolol on POD 6. By POD 2, the bleeding in the chest tube had completely subsided, and the heparin infusion was restarted and the oral aspirin started per protocol. After the first week, the arterial line and chest tube was removed, and the recipient was monitored via daily lab draws and weekly sedations. During these planned sedations, echocardiograms, weight, and physical examinations were taken, and every other week the subject was taken for right heart catheterization. Approximately 4 weeks post-transplant, the white blood cell count had increased along with the recipient appearing slightly lethargic, and we suspected this to be the result of a central line infection, which was treated with antibiotics (linezolid, 15 mg/kg and meropenem, 20 mg/kg) and an antifungal (micafungin, 3 mg/kg). The maintenance immunosuppression (MMF) was held for 48 h and then lowered to 40 mg/kg/day from 110 mg/kg/day for one week, which resulted in clinical improvement in the subject. The recipient expired on postoperative day 55, likely due to AMR secondary to unplanned weaning of immunosuppression for suspected infection.

**Figure 6:**
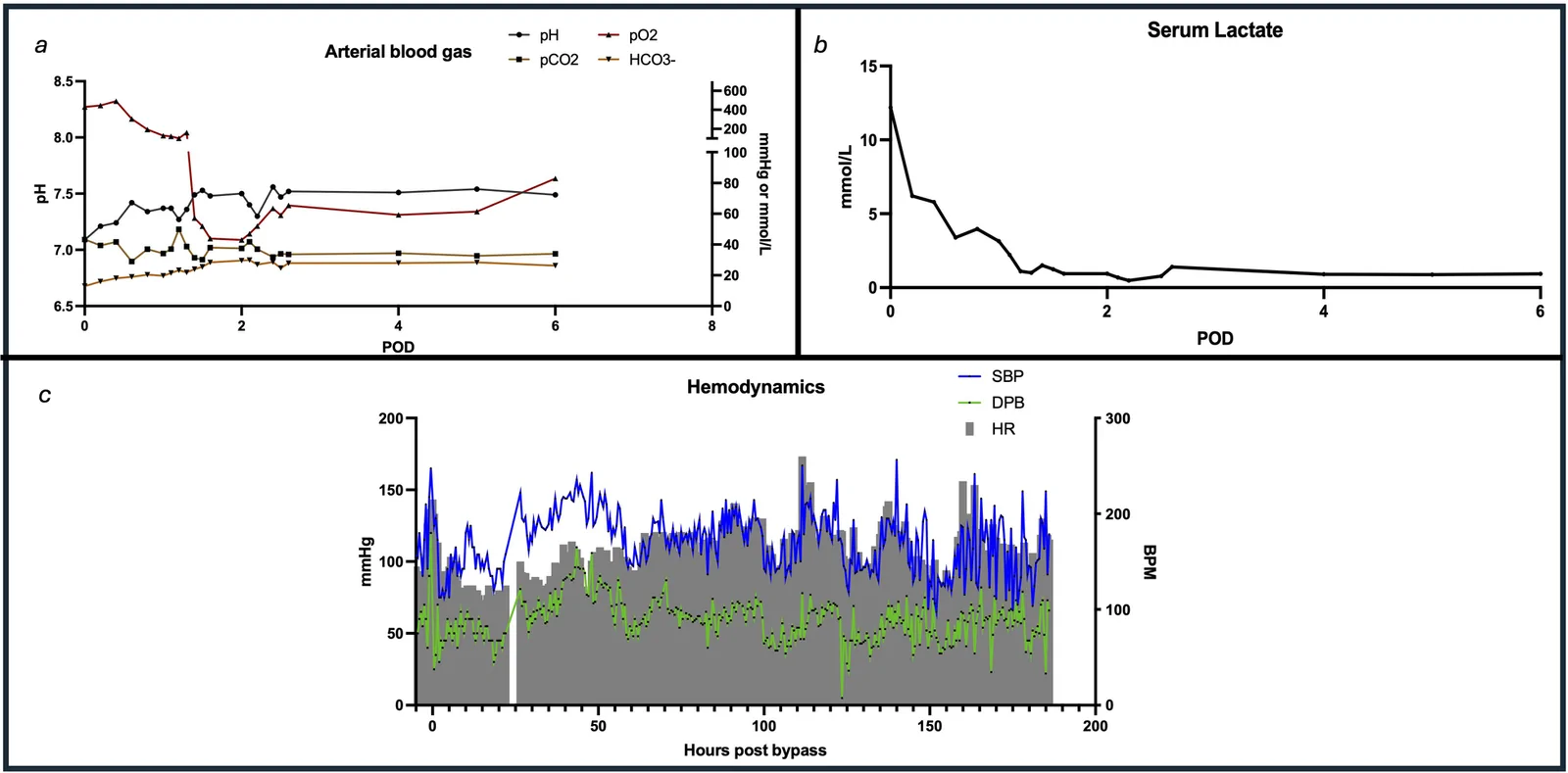
Postoperative clinical data.

### Echocardiography

The results of the echocardiogram from postoperative day 0 and 1 are described above. The next echocardiogram was performed 8 days later and at least every week or sooner depending on the clinical condition of the animal. By POD 8, the echocardiogram had improved, with the LVEF increasing to 55.9%, the LV fractional shortening increasing to 20.2%, significant improvement in the MR and TR. Echocardiograms performed on POD 14 and 21 demonstrated stable function of the xenograft. The echo performed on POD 29 demonstrated the first signs of dysfunction, with the LVEF decreasing to 43.9%, LV fractional shortening to 18.8%, although stable MR and TR from previous studies. By POD 42, LV function continued to decline, with an EF of 38% (Figure 7A).

**Figure 7:**
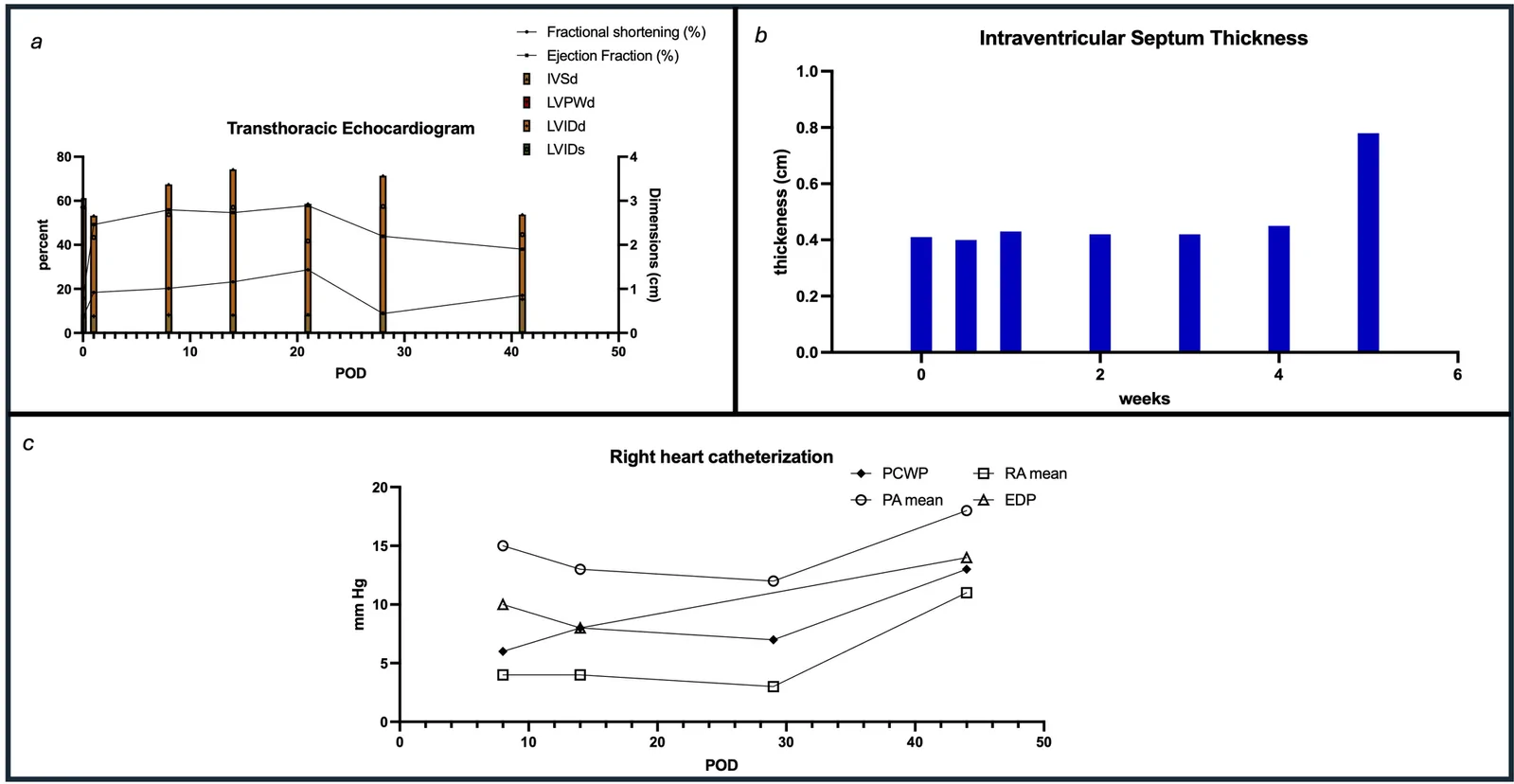
Postoperative echo and RHC data.

It was also observed that on POD 21, a mass in the apex of the left ventricle, approximately at the location of a cannula site during the *ex vivo* perfusion of the xenograft (Figure 8). On POD 29, the mass appeared to be similar in size, although more mobile. The final echocardiogram on POD 42 noted the mass in the LV to be slightly smaller in size and less mobile. It is hypothesized that the cannula insertion caused an injury at the LV apex, although histological examination was inconclusive. Due to the concern for rapid growth of the xenograft which has been described by other groups investigating cardiac xenotransplantation (32), we closely monitored for graft growth by measuring the thickness of the intraventricular septum (IVS) (Figure 7B). For the first several weeks post-transplant, there was essentially no change in the IVS thickness. At the point of the last echocardiogram on POD 42, the IVS thickness had increased from around 0.4–0.8 cm, which we suspect was due to AMR in the setting of significantly lowering the immunosuppression to treat a central line infection.

**Figure 8:**
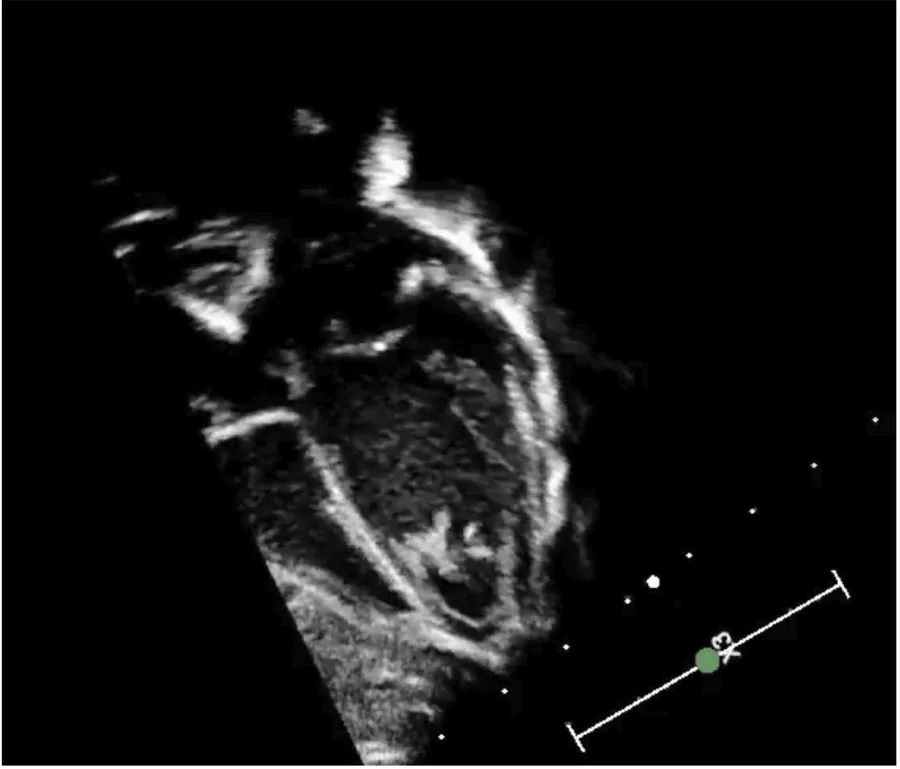
Echocardiogram on POD 21 demonstrating LV mass.

### Right heart catheterization

RHC were performed on POD 8, 14, 28, and 44 via the techniques described above, successfully and without technical issues. The hemodynamics measured during this invasive intervention demonstrated stable wedge pressures, RV pressures, PA pressures, RA pressures, and end-diastolic pressures until POD 44 (Figure 7C).

### Pathology

Tissue was obtained for histological analysis via the RHC on postoperative days 8, 14, 28, 44, and 55 at the time of the necropsy (Figure 9). The hematoxylin and eosin (H&E) and C4d staining demonstrated mild edema and minimal interstitial hemorrhage, but no evidence of active AMR, and there was no evidence of thrombotic microangiopathy (TMA). By postoperative day 28, C4d was focally positive, with mild edema and no evidence of TMA, although there was one focus of mononuclear infiltrate and focal myocytes at the periphery of tissue showing ischemic or early necrotic changes. This sample was taken several days after suspending the dose of maintenance immunosuppression (MMF) for 48 h and then lowering the dose to 40 mg/kg/day from 110 mg/kg/day for a suspected line infection for one week. By postoperative day 44, histological features of AMR were noted, and C4d staining was focally positive along <50% of capillaries, not meeting criteria for immunopathologic evidence of AMR (33, 34). The animal died on postoperative day 55, likely secondary to xenograft AMR induced by primary systemic line infection and unplanned suspension/lowering of maintenance immunosuppression. Histologically, evidence of acute endothelial/vascular injury secondary to AMR, although no evidence of TMA. No evidence of acute cellular rejection was noted.

**Figure 9:**
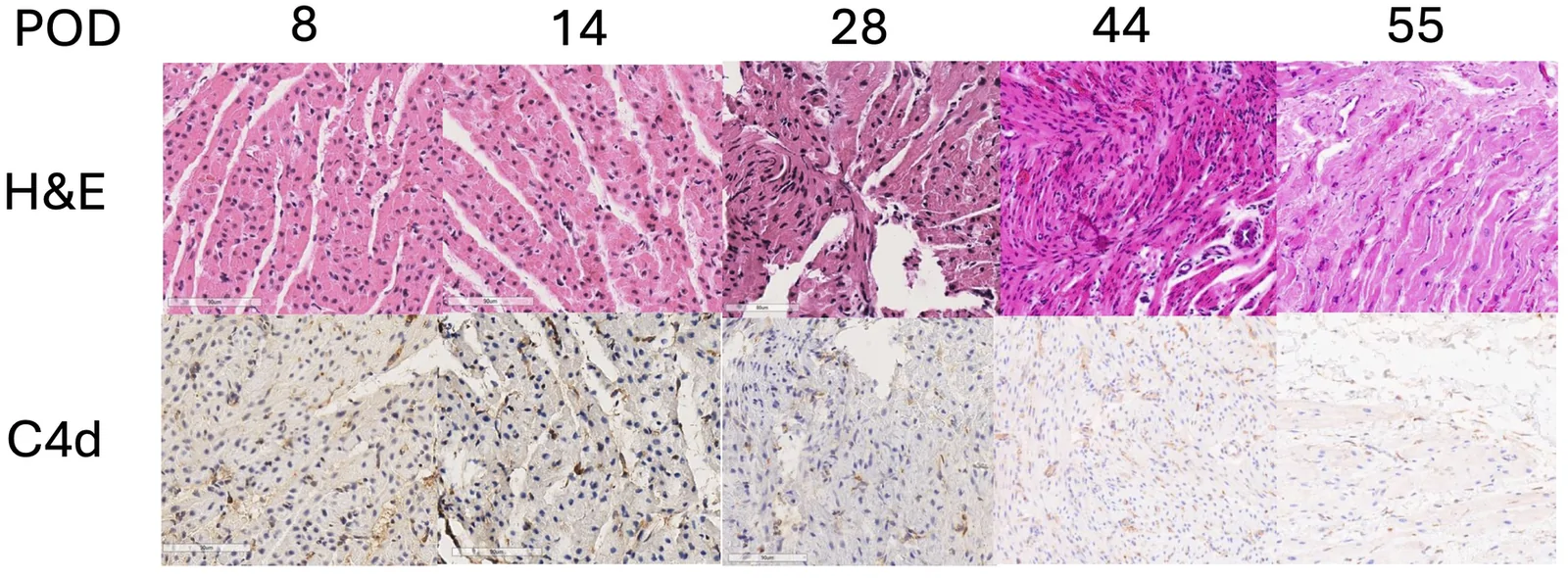
Pathology*.*

## Discussion

Our study demonstrates the successful development of a novel model to study orthotopic cardiac xenotransplantation in NHPs utilizing our novel nonischemic, normothermic, beating heart xenotransplant technique. The goal of this study was to demonstrate the feasibility of eliminating IR injury in cardiac xenografts, which has never previously been achieved. We hypothesize that by eliminating all ischemic time and keeping the heart normothermic and beating during the explant, transport, and implant process, rates of PCXD will be significantly reduced or eliminated outright. Additional studies will need to be performed utilizing this technique to investigate if this approach results in improved survival in cardiac xenotransplantation. Early reports in human-to-human heart transplant utilizing a similar beating heart technique have demonstrated good results, although long-term outcomes have yet to be reported (35, 36).

*Ex vivo* coronary perfusion without using cardioplegia to arrest the heart, and without proprietary, costly *ex vivo* organ perfusion machines, is technically feasible and facilitates excellent early graft function. Our study also demonstrates that a single gene edited porcine heart donor can produce early graft survival without rejection or other immunological complications. The field of cardiac xenotransplantation has focused on using a maximum genetic engineering approach for their porcine donors, assuming many genetic edits are essential for survival, yet we achieved this excellent outcome with a single gene edit, favoring the hypothesis that non-ischemic transplantation could be beneficial in cardiac xenotransplantation utilizing a single-gene edited donor. This ‘never ischemic’ beating heart transplant technique not only has important implications for bringing cardiac xenotransplantation to the clinic but also has broader translatability in allotransplantation.

A major mechanism contributing to the limited length of survival in cardiac xenotransplantation is believed to be related to the IR injury associated with the traditional methods of heart procurement and implantation (37). It has been described in allogeneic heart transplantation that IR injury has a major role in immediate graft dysfunction and chronic graft rejection (38). This process disrupts the electron transport chain, which leads to increased intracellular calcium and reactive oxygen species formation, subsequently leading to mitochondrial membrane rupture and cell death. Cell death leads to a spillage of numerous cellular components from the cardiomyocytes, termed damage-associated molecular patterns (DAMPs), which serve as ligands for pattern recognition receptors for the innate immune system. This leads to a release of pro-inflammatory cytokines in the recipient, and subsequent allograft injury (39). This process is likely more injurious in cardiac xenotransplantation as the cross species exposure of porcine DAMPs to a primate's immune system can be expected to produce a more dramatic activation of the immune system compared to an allograft (18). We can conclude this based on NHP experiments which demonstrated that even in the presence of a relatively short length of time of cold ischemia with similar preservation techniques, which would be acceptable in allogeneic heart transplantation, PCXD ensues and leads to death (21).

To reduce the IR injury encountered in these experiments, we developed a novel technique of harvesting and implanting a genetically modified porcine heart without an obligatory period of ischemia encountered in traditional heart procurement. Our technique eliminates the period of ischemia compared to all other methods, including the use of *ex vivo* perfusion devices (40). The technique is described above, along with the methods of selecting an appropriate recipient, conducting postoperative animal care, and evaluation of the xenograft in the postoperative period. While our lab is currently conducting experiments in several consecutive NHP animals to understand how this novel method may improve long-term survival in cardiac xenotransplantation to reduce primary xenograft dysfunction, the purpose of this manuscript is to describe the technique and demonstrate the feasibility by presenting the short-term results in a limited cohort of experiments. Our long-term results will be published in a subsequent manuscript. Briefly, our study successfully demonstrated the ability to perform an orthotopic heart transplant from a miniature *GGTA1* null piglet into a juvenile nonhuman primate while keeping the donor heart beating at normothermia during the entire explant, transport, and implant period. Our technique facilitated separating the NHP recipient from cardiopulmonary bypass on minimal inotropes, extubated within 24 h, and returned to the standard housing unit for NHPs.

An additional area warranting further investigation is recipient exposure to porcine blood. During the performance of our transplant experiment, an undetermined volume of donor porcine whole blood was transferred with the donor heart into the nonhuman primate recipient, potentially eliciting both innate and adaptive immune responses. Historical experience with xenotransfusion—dating from unsuccessful attempts in the mid-19th century prior to the identification of ABO blood groups—demonstrates that wild-type porcine blood is immunogenic in primates (41). Although the use of *GGTA1* null donor pigs mitigates a principal target of antibody-mediated injury by eliminating the Gal antigen, other xenoantigens capable of triggering antibody-dependent cytotoxicity remain (42). In addition, interspecies incompatibilities in complement and coagulation regulatory pathways may further amplify immune activation (43). The immunologic consequences of transient exposure to *GGTA1* null porcine blood in this setting remain undefined and may represent an underappreciated contributor to recipient immune activation.

In conclusion, cardiac xenotransplantation is on the cusp of clinical translatability. Eliminating ischemia-reperfusion injury in cardiac xenotransplantation has the potential to significantly increase the length of survival after orthotopic porcine heart transplantation by eliminating rates of PCXD. This in turn could bring cardiac xenotransplantation to the clinic and significantly reduce the burden for those in need of a heart transplant. The pioneering cardiac transplant surgeon Dr. Shumway once stated that “xenotransplantation is the future of transplantation and always will be” (44). While Dr. Shumway's statement highlights the potential of xenotransplantation to dramatically alter the field of transplantation, his statement failed to anticipate the advancements in genetic engineering and novel immunosuppression which would bring this field on the brink of clinical translatability.

## Data Availability

The raw data supporting the conclusions of this article will be made available by the authors, without undue reservation.

## References

[1] Kazi DS Elkind MSV Deutsch A Dowd WN Heidenreich P Khavjou O. Forecasting the economic burden of cardiovascular disease and stroke in the United States through 2050: a presidential advisory from the American Heart Association. Circulation. (2024) 150:e89–e101. 10.1161/CIR.0000000000001258 38832515

[2] Jou S Mendez SR Feinman J Mitrani LR Fuster V Mangiola M. Heart transplantation: advances in expanding the donor pool and xenotransplantation. Nat Rev Cardiol. (2024) 21:25–36. 10.1038/s41569-023-00902-1 37452122

[3] Peng DM Koehl DA Cantor RS McMillan KN Barnes AP McConnell PI. Outcomes of children with congenital heart disease implanted with ventricular assist devices: an analysis of the pediatric interagency registry for mechanical circulatory support (pedimacs). J Heart Lung Transplant. (2019) 38:420–30. 10.1016/j.healun.2018.10.008 30459063 PMC6449204

[4] Butts RJ Toombs L Kirklin JK Schumacher KR Conway J West SC. Waitlist outcomes for pediatric heart transplantation in the current era: an analysis of the pediatric heart transplant society database. Circulation. (2024) 150:362–73. 10.1161/CIRCULATIONAHA.123.068189 38939965

[5] Woolley AE Singh SK Goldberg HJ Mallidi HR Givertz MM Mehra MR. Heart and lung transplants from HCV-infected donors to uninfected recipients. N Engl J Med. (2019) 380:1606–17. 10.1056/NEJMoa1812406 30946553 PMC7369135

[6] Schroder JN Patel CB DeVore AD Bryner BS Casalinova S Shah A. Transplantation outcomes with donor hearts after circulatory death. N Engl J Med. (2023) 388:2121–31. 10.1056/NEJMoa2212438 37285526

[7] Sykes M Sachs DH. Progress in xenotransplantation: overcoming immune barriers. Nat Rev Nephrol. (2022) 18:745–61. 10.1038/s41581-022-00624-6 36198911 PMC9671854

[8] Wolbrom DH Kim JI Griesemer A. The road to xenotransplantation. Curr Opin Organ Transplant. (2023) 28:65–70. 10.1097/MOT.0000000000001055 36752272 PMC11323750

[9] Phillips KG Aljabban I Wolbrom DH Griesemer A Leacche M McGregor C. Cardiac xenotransplantation: current state and future directions. Circulation. (2025) 152:58–73. 10.1161/CIRCULATIONAHA.124.070875 40623074

[10] Jinek M Chylinski K Fonfara I Hauer M Doudna JA Charpentier E. A programmable dual-RNA-guided DNA endonuclease in adaptive bacterial immunity. Science. (2012) 337:816–21. 10.1126/science.1225829 22745249 PMC6286148

[11] Mohiuddin MM Singh AK Corcoran PC Hoyt RF Thomas ML Lewis BGT. Role of anti-CD40 antibody-mediated costimulation blockade on non-gal antibody production and heterotopic cardiac xenograft survival in a GTKO.hCD46Tg pig-to-baboon model. Xenotransplantation. (2014) 21:35–45. 10.1111/xen.12066 24164510 PMC5603077

[12] Adams AB Lovasik BP Faber DA Burlak C Breeden C Estrada JL. Anti-C5 antibody tesidolumab reduces early antibody-mediated rejection and prolongs survival in renal xenotransplantation. Ann Surg. (2021) 274:473–80. 10.1097/SLA.0000000000004996 34238812 PMC8915445

[13] Miller CL Madsen JC. Targeting IL-6 to prevent cardiac allograft rejection. Am J Transplant. (2022) 22(Suppl 4):12–7. 10.1111/ajt.17206 36453706 PMC10191185

[14] Mohiuddin MM Singh AK Scobie L Goerlich CE Grazioli A Saharia K. Graft dysfunction in compassionate use of genetically engineered pig-to-human cardiac xenotransplantation: a case report. Lancet. (2023) 402:397–410. 10.1016/S0140-6736(23)00775-4 37393920 PMC10552929

[15] DiChiacchio L Singh AK Lewis B Zhang T Hardy N Pasrija C. Early experience with preclinical perioperative cardiac xenograft dysfunction in a single program. Ann Thorac Surg. (2020) 109:1357–61. 10.1016/j.athoracsur.2019.08.090 31589847 PMC8459574

[16] Laubach VE Sharma AK. Mechanisms of lung ischemia-reperfusion injury. Curr Opin Organ Transplant. (2016) 21:246–52. 10.1097/MOT.0000000000000304 26945320 PMC4861054

[17] Salvadori M Rosso G Bertoni E. Update on ischemia-reperfusion injury in kidney transplantation: pathogenesis and treatment. World J Transplant. (2015) 5:52–67. 10.5500/wjt.v5.i2.52 26131407 PMC4478600

[18] Patel PM Connolly MR Coe TM Calhoun A Pollok F Markmann JF. Minimizing ischemia reperfusion injury in xenotransplantation. Front Immunol. (2021) 12:681504. 10.3389/fimmu.2021.681504 34566955 PMC8458821

[19] Byrne GW McGregor CG. Cardiac xenotransplantation: progress and challenges. Curr Opin Organ Transplant. (2012) 17:148–54. 10.1097/MOT.0b013e3283509120 22327911 PMC3326642

[20] Byrne GW Du Z Sun Z Asmann YW McGregor CG. Changes in cardiac gene expression after pig-to-primate orthotopic xenotransplantation. Xenotransplantation. (2011) 18:14–27. 10.1111/j.1399-3089.2010.00620.x 21342284 PMC10022692

[21] Längin M Reichart B Steen S Sjöberg T Paskevicius A Liao Q. Cold non-ischemic heart preservation with continuous perfusion prevents early graft failure in orthotopic pig-to-baboon xenotransplantation. Xenotransplantation. (2021) 28:e12636. 10.1111/xen.12636 32841431

[22] Mohiuddin MM Goerlich CE Singh AK Zhang T Tatarov I Lewis B. Progressive genetic modifications of porcine cardiac xenografts extend survival to 9 months. Xenotransplantation. (2022) 29:e12744. 10.1111/xen.12744 35357044 PMC10325874

[23] Cleveland JD Mitchell CB Swicord W Neal SJ Vo C Bakshi K. Gene-edited pig cardiac xenotransplantation as a bridge to allotransplantation in infants: progress in a pig-to-baboon model. Am J Transplant. (2026) 26:980–91. 10.1016/j.ajt.2025.12.017 41453738 PMC12935157

[24] Längin M Mayr T Reichart B Michel S Buchholz S Guethoff S. Consistent success in life-supporting porcine cardiac xenotransplantation. Nature. (2018) 564:430–3. 10.1038/s41586-018-0765-z 30518863

[25] Tasaki M Wamala I Tena A Villani V Sekijima M Pathiraja V. High incidence of xenogenic bone marrow engraftment in pig-to-baboon intra-bone bone marrow transplantation. Am J Transplant. (2015) 15:974–83. 10.1111/ajt.13070 25676635 PMC4407988

[26] Watanabe H Sahara H Nomura S Tanabe T Ekanayake-Alper DK Boyd LK. GalT-KO pig lungs are highly susceptible to acute vascular rejection in baboons, which may be mitigated by transgenic expression of hCD47 on porcine blood vessels. Xenotransplantation. (2018) 25:e12391. 10.1111/xen.12391 29527745 PMC6135720

[27] Hussain ST Kumar A Chan J James L Smith D Moazami N. Anatomical considerations and surgical technique of porcine cardiac xenotransplantation. JTCVS Tech. (2024) 25:104–10. 10.1016/j.xjtc.2024.03.019 38899090 PMC11184667

[28] Lopez L Saurers DL Barker PCA Cohen MS Colan SD Dwyer J. Guidelines for performing a comprehensive pediatric transthoracic echocardiogram: recommendations from the American society of echocardiography. J Am Soc Echocardiogr. (2024) 37:119–70. 10.1016/j.echo.2023.11.015 38309834

[29] Shah A Goerlich CE Pasrija C Hirsch J Fisher S Odonkor P. Anatomical differences between human and pig hearts and their relevance for cardiac xenotransplantation surgical technique. JACC Case Rep. (2022) 4:1049–52. 10.1016/j.jaccas.2022.06.011 36062051 PMC9434648

[30] Längin M Konrad M Reichart B Mayr T Vandewiele S Postrach J. Hemodynamic evaluation of anesthetized baboons and piglets by transpulmonary thermodilution: normal values and interspecies differences with respect to xenotransplantation. Xenotransplantation. (2020) 27:e12576. 10.1111/xen.12576 31854004

[31] Mayr T Bauer A Reichart B Guethoff S Schoenmann U Längin M. Hemodynamic and perioperative management in two different preclinical pig-to-baboon cardiac xenotransplantation models. Xenotransplantation. (2017) 24:e12295. 10.1111/xen.12295 28261878

[32] Bender M Panelli A Reichart B Radan J Mokelke M Neumann E. Hemodynamics in pig-to-baboon heterotopic thoracic cardiac xenotransplantation: recovery from perioperative cardiac xenograft dysfunction and impairment by cardiac overgrowth. Xenotransplantation. (2024) 31:e12841. 10.1111/xen.12841 38864375 PMC11167678

[33] Berry GJ Burke MM Andersen C Bruneval P Fedrigo M Fishbein MC. The 2013 international society for heart and lung transplantation working formulation for the standardization of nomenclature in the pathologic diagnosis of antibody-mediated rejection in heart transplantation. J Heart Lung Transplant. (2013) 32:1147–62. 10.1016/j.healun.2013.08.011 24263017

[34] Kobashigawa J Colvin M Potena L Dragun D Crespo-Leiro MG Delgado JF. The management of antibodies in heart transplantation: an ISHLT consensus document. J Heart Lung Transplant. (2018) 37:537–47. 10.1016/j.healun.2018.01.1291 29452978

[35] Krishnan A Kasinpila P Wang H Ruaengsri C Shudo Y Jackson E. First-in-human beating-heart transplant. JTCVS Tech. (2023) 19:80–5. 10.1016/j.xjtc.2023.02.015 37324334 PMC10267812

[36] Yin S Rong J Chen Y Cao L Liu Y Mo S. Transplantation of a beating heart: a first in man. Lancet Reg Health West Pac. (2022) 23:100449. 10.1016/j.lanwpc.2022.100449 35465045 PMC9019404

[37] Shumway NE Lower RR Stofer RC. Transplantation of the heart. Adv Surg. (1966) 2:265–84. 5333297

[38] Dashkevich A Raissadati A Syrjälä SO Zarkada G Keränen MAI Tuuminen R. Ischemia-reperfusion injury enhances lymphatic endothelial VEGFR3 and rejection in cardiac allografts. Am J Transplant. (2016) 16:1160–72. 10.1111/ajt.13564 26689983

[39] Silvis MJM Kaffka genaamd Dengler SE Odille CA Mishra M Van Der Kaaij NP Doevendans PA. Damage-associated molecular patterns in myocardial infarction and heart transplantation: the road to translational success. Front Immunol. (2020) 11:599511. 10.3389/fimmu.2020.599511 33363540 PMC7752942

[40] Bryner BS Schroder JN Milano CA. Heart transplant advances: *ex vivo* organ-preservation systems. JTCVS Open. (2021) 8:123–7. 10.1016/j.xjon.2021.04.020 36004090 PMC9390583

[41] Roux FA Saï P Deschamps JY. Xenotransfusions, past and present. Xenotransplantation. (2007) 14:208–16. 10.1111/j.1399-3089.2007.00404.x 17489860

[42] Chornenkyy Y Yamamoto T Hara H Stowell SR Ghiran I Robson SC. Future prospects for the clinical transfusion of pig red blood cells. Blood Rev. (2023) 61:101113. 10.1016/j.blre.2023.101113 37474379 PMC10968389

[43] Maenaka A Terashita M Kinoshita K Cooper DKC. Human CD55 expression and C1 inhibition partially protect gene-edited pig red blood cells from human complement-mediated hemolysis *in vitro*. Xenotransplantation. (2025) 32:e70060. 10.1111/xen.70060 40492338

[44] Reichart B Guethoff S Mayr T Thormann M Buchholz S Postrach J. Discordant cardiac xenotransplantation: broadening the horizons. Eur J Cardiothorac Surg. (2014) 45:1–5. 10.1093/ejcts/ezt483 24096411

